# Inhabitancy of active *Nitrosopumilus*-like ammonia-oxidizing archaea and *Nitrospira* nitrite-oxidizing bacteria in the sponge *Theonella swinhoei*

**DOI:** 10.1038/srep24966

**Published:** 2016-04-26

**Authors:** Guofang Feng, Wei Sun, Fengli Zhang, Loganathan Karthik, Zhiyong Li

**Affiliations:** 1 State Key Laboratory of Microbial Metabolism, and School of Life Sciences & Biotechnology, Shanghai Jiao Tong University, Shanghai 200240, China

## Abstract

Nitrification directly contributes to the ammonia removal in sponges, and it plays an indispensable role in sponge-mediated nitrogen cycle. Previous studies have demonstrated genomic evidences of nitrifying lineages in the sponge *Theonella swinhoei*. However, little is known about the transcriptional activity of nitrifying community in this sponge. In this study, combined DNA- and transcript-based analyses were performed to reveal the composition and transcriptional activity of the nitrifiers in *T. swinhoei* from the South China Sea. Transcriptional activity of ammonia-oxidizing archaea (AOA) and nitrite-oxidizing bacteria (NOB) in this sponge were confirmed by targeting their nitrifying genes,16S rRNA genes and their transcripts. Phylogenetic analysis coupled with RDP rRNA classification indicated that archaeal 16S rRNA genes, *amoA* (the subunit of ammonia monooxygenase) genes and their transcripts were closely related to *Nitrosopumilus*-like AOA; whereas nitrifying bacterial 16S rRNA genes, *nxrB* (the subunit of nitrite oxidoreductase) genes and their transcripts were closely related to *Nitrospira* NOB. Quantitative assessment demonstrated relative higher abundances of nitrifying genes and transcripts of *Nitrosopumilus*-like AOA than those of *Nitrospira* NOB in this sponge. This study illustrated the transcriptional potentials of *Nitrosopumilus*-like archaea and *Nitrospira* bacteria that would predominantly contribute to the nitrification functionality in the South China Sea *T. swinhoei*.

The marine nitrogen cycle controls the availability of nitrogenous nutrients and the biological productivity in oceanic systems^1^. The nitrogen cycle consists of multiple transformations of nitrogenous compounds, primarily driven by microbes. One key link of this cycle is the nitrification process, which occurs in many marine ecosystems, such as seawaters, sediments, hydrothermal vents and in invertebrates like sponges^2^,^3^,^4^,^5^.

Nitrification describes the oxidation of ammonia to nitrite and subsequently to nitrate for energy purposes^6^. Ammonia-oxidizing archaea (AOA) and bacteria (AOB) perform the first, often rate-determining step of nitrification from ammonia to nitrite, while nitrite-oxidizing bacteria (NOB) are responsible for the oxidation of nitrite to nitrate. Nearly all the reported AOA are ascribed into the phylum Thaumarchaeota, and AOB are affiliated with some genera of the phylum Proteobacteria (e.g. *Nitrosomonas*, *Nitrosococcus*, *Nitrosospira*, *Nitrosovibrio*); while all known NOB belong to some genera of the phyla Proteobacteria (e.g. *Nitrobacter*, *Nitrococcus*, *Nitrotoga*), Nitrospinae (e.g. *Nitrospina*), Nitrospirae (e.g. *Nitrospira*) and Chloroflexi (e.g. *Nitrolancea*)^7^. In view of their phylogenetic diversity, the 16S rRNA gene, *amoA* gene (encoding the subunit of the ammonia monooxygenase in AOA and AOB) and *nxrA* or *nxrB* gene (encoding the subunit of the nitrite oxidoreductase in NOB) have served as useful phylogenetic markers for investigating nitrifying communities in various ecosystems^8^,^9^,^10^,^11^,^12^,^13^,^14^,^15^.

Sponges are sessile invertebrates that grow widely in virtually all aquatic ecosystems with high filtration rates^16^. Many sponges harbor phylogenetically complex microbial assemblages including bacteria, archaea, fungi and microalgae^17^. For example, surveys of sponge symbiotic communities have revealed no less than 47 bacterial phyla and two archaeal phyla so far^18^. However, compared with the knowledge about the diversity of sponge microbial symbionts, our understanding of their functions is still limited^19^. Given the overall importance of ecological functions of symbionts to the sponge holobionts, nitrogen metabolism in sponges is continuing to receive considerable attention, since nitrogen is generally a limiting factor in the oligotrophic habitat of sponges.

Like many other marine invertebrates, sponges excrete ammonia as a metabolic waste^20^. Nitrification process that contributes to ammonia removal in sponges has been suggested by using incubation experiments^20^,^21^. In sponges, nitrifiers have been corroborated by the isolation of individual species^22^,^23^, and by the identification of 16S rRNA genes or functional genes of nitrifying microbes from sponge microbiomes by using molecular-based approaches. For example, diverse *Nitrosospira* AOB and Planctomycetes anaerobic bacteria in the sponges *Ircinia strobilina* and *Mycale laxissima* were confirmed by *amoA* gene and 16SrRNA gene assays^24^. Meanwhile, vertical transmission of AOA from mother to offspring was suggested in a diverse range of sponges from different biogeographic areas by 16S rRNA gene and *amoA* gene detection results^5^. Moreover, the increasing gene-targeted and shotgun high-throughput sequencing technologies have strongly illustrated the ecological potentials of sponge microbes, although many limitations persist^25^. For example, 16S rRNA-targeted pyrosequencing analysis has revealed the *Nitrosopumilus* lineage in the sponge holobionts^26^. Meanwhile, shotgun metagenomic analysis of the sponge *Neamphius huxleyi* has demonstrated the genomic potentials, including *Nitrosopumilus* ammonia oxidation, at the functional-population level^27^. Recent shotgun metagenomic sequencing analysis of the sponges *Didiscus oxeata* and *Scopalina ruetzleri* has uncovered the symbionts’ functions at the whole-community level, including the nitrification process that might be performed by a diverse set of archaeal and bacterial nitrifying lineages, including *Nitrosopumilus*, *Nitrosococcus*, *Nitrosomonas* and *Nitrosospira*^28^, suggesting the presence of various nitrifying microbes, e.g. AOA and AOB in sponges.

In many niches, such as sponges, the presence of functional genes is not the direct evidence of their corresponding metabolic activity *in situ*, whereas RNA-based strategies would provide valuable hints on the activity of microbial assemblage^29^, albeit imprecisely in particular cases^30^. To date, a number of RNA-based analyses have been performed in revealing the activity of microbial communities in sponges in their natural physiological state or under certain environmental stresses^31^,^32^, and in conjecturing the active microbial metabolic processes, such as nitrogen fixation, ammonia oxidation or urea hydrolysis in sponges^33^,^34^,^35^. Moreover, metatranscriptomic analysis has been performed to give a whole-community-level exploration of the *in situ* active functions, such as archaeal ammonia oxidation and photosynthetic carbon fixation, carried out by sponge symbionts^36^,^37^,^38^. However, functional gene-targeted high-throughput sequencing of interested functional genes, such as *amoA* and *nxrA*/*nxrB* genes, has not been performed in sponge metagenome/metatranscriptome so far.

The sponge *Theonella swinhoei* (class Demospongiae, order Lithistida, family Theonellidae) is widely distributed in tropical and subtropical oceans^39^. Previous DNA-based investigations have demonstrated the nitrifying potential of the *Nitrosopumilus*, *Nitrosospira* and *Nitrospira* lineages in *T. swinhoei* near Caroline Islands, Palau (Palauan *T. swinhoei*)^21^,^40^. However, little is known about the transcriptional activity of nitrifiers in this sponge. Hence, the present study aims to reveal the community structure and transcriptional activity of nitrifiers in *T. swinhoei* from the South China Sea using a DNA and RNA combined approach targeting the nitrifying genes, 16S rRNA genes and their transcripts.

## Results

### Amplification of 16S rRNA, *amoA* and *nxrA*/*nxrB*

The archaea and bacteria were detected by PCR amplification using the16S rRNA primers (Table 1) and nitrifiers were verified using the primer pairs (Table 1) targeting *amoA*, *nxrA* and *nxrB* genes from *T. swinhoei* DNA and cDNA templates. Cloning and sequencing results showed that archaeal and bacterial 16S rRNA gene, archaeal *amoA* gene and *Nitrospira nxrB* gene were positively amplified from both DNA and cDNA templates; while bacterial *amoA* gene, *Nitrobacter nxrA* gene, *Nitrococcus nxrA* gene, *Nitrolancea nxrA* gene and *Nitrospina nxrB* gene were not detected from either DNA or cDNA templates (data not shown). After quality check and trimming, archaeal 16S rRNA fragments of 657 ± 3-bp length, bacterial 16S rRNA fragments of 694 ± 14-bp length, archaeal *amoA* fragments of 532-bp length and *Nitrospira nxrB* fragments of 469-bp length were used for further analysis. Rarefaction curve analysis showed that the amount of sequenced clones in each clone library, shown in Table 1, reached saturation at 95% or 97% sequence similarity (Supplementary Fig. S1).

### Active archaea and AOA in the South China Sea *T. swinhoei*

**Figure 1:**
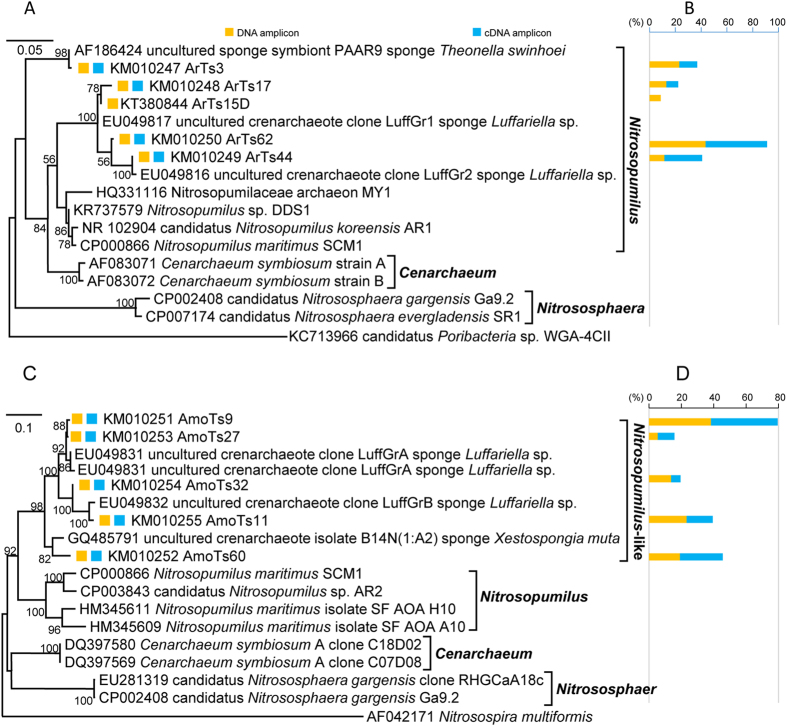
Maximum-likelihood phylogenetic analysis of archaeal 16S rRNA genes and transcripts (**A**) and the percentage of each OTU (97% sequence similarity) in the corresponding clone library (**B**), and maximum-likelihood phylogenetic analysis of archaeal *amoA* genes and transcripts (**C**) and the percentage of each OTU (95% sequence similarity) in the corresponding clone library (**D**) from the South China Sea *T. swinhoei*. OTU representatives are marked. Scale bar represents 5% (for 16S rRNA) or 10% (for *amoA*) nucleotide sequence divergence per homologues position. Bootstrap values more than 50% of 1000 replicates are shown on the trees. The outgroup of the archaeal 16S rRNA tree is a bacterial 16S rRNA sequence of candidatus *Poribacteria* sp. WGA-4CII (accession no. KC713966) and the outgroup of the archaeal *amoA* tree is a bacterial *amoA* sequence of *Nitrosospira multiformis* (accession no. AF042171).

Composition and activity of archaea in the South China Sea *T. swinhoei* was detected by 16S rRNA gene and transcript amplification. The 69 16S rRNA gene amplicons and 65 16S rRNA transcript amplicons were grouped into five OTUs (accession no. KM010247–KM010250 and KT380844) belonging to the *Nitrosopumilus* cluster, according to phylogenetic analysis and RDP Classifier identification (Fig. 1A; Supplementary Table S1). Four of these OTUs were shared at DNA/transcript levels, while the left one was unique at the DNA level (Supplementary Table S3). All the OTUs showed 94–99% similarity to the sequences from the sponge *Luffariella* sp. (accession no. EU049816 and EU049817) and *T. swinhoei* (accession no. AF186424); meanwhile, these OTUs shared 93–94% identity with the sequences of *Nitrosopumilus* spp. (accession no. KR737579 and HQ331116) and 90–92% identity with the sequences of *Cenarchaeum* spp. (accession no. AF083071 and AF083072). Each OTU took different proportions of the sequences in the corresponding clone library ranging from 9–48% (Fig. 1B). Thus, our findings suggested that *Nitrosopumilus* species dominated the archaeal community in the South China Sea *T. swinhoei*, and a large fraction of archaea had transcriptional activity in this sponge host.

Furthermore, PCR assays of archaeal *amoA* genes and transcripts verified the presence of active ammonia-oxidizing population in the South China Sea *T. swinhoei*. All the amplicons of archaeal *amoA* genes and transcripts were assorted to five shared OTUs at DNA/transcript levels (accession no. KM010251–KM010255) (Fig. 1C). Nucleic acid sequences rather than deduced amino acid sequences were used for phylogeny reconstruction, as the former shows higher phylogenetic resolution. Each *amoA* OTU accounted for 5–41% of the sequences in the respective clone library (Fig. 1D). These OTUs were closely (93–99% sequence identity) related to the sequences from the sponges *Luffariella* sp. (accession no. EU049831- EU049833) and *Xestospongia muta* (accession no. GQ485791). Meanwhile, these OTUs showed 79–81% similarity with the *amoA* sequences of *Nitrosopumilus* spp. (accession no. CP000866, CP003843, HM345611 and HM345609) and 77–79% similarity with the *amoA* sequences of *Cenarchaeum* spp. (accession no. DQ397569 and DQ397580) (Supplementary Table S2). On the peptide level, these OTUs shared 94–96% similarity with the AmoA sequences of *Nitrosopumilus* spp. (accession no. CP000866, CP003843, HM345611 and HM345609) and 92–93% similarity with the AmoA sequences of *Cenarchaeum* spp. (accession no. DQ397569 and DQ397580), which indicated that they were more likely the *Nitrosopumilus*-like *amoA* sequences.

Altogether, the phylogeny of archaeal 16S rRNA genes and transcripts strongly demonstrated that the active archaeal community was dominated by *Nitrosopumilus* species in the South China Sea *T. swinhoei*; particularly, *Nitrosopumilus*-like AOA with transcriptional activity might predominantly contribute to the aerobic ammonia oxidation in this sponge.

### Active bacteria and NOB in the South China Sea *T. swinhoei*

**Figure 2:**
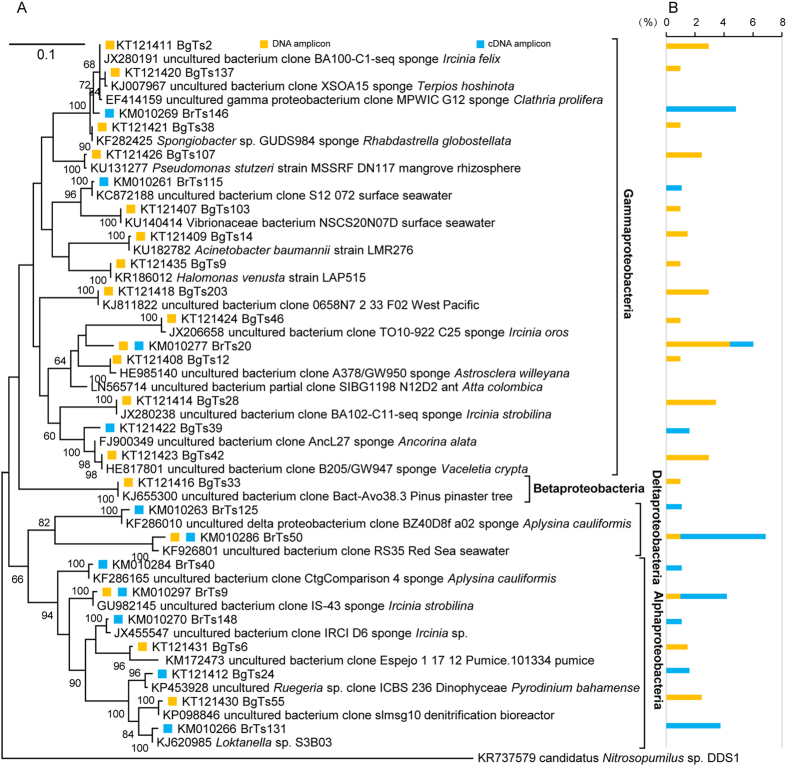
Maximum-likelihood phylogenetic analysis of Proteobacteria 16S rRNA genes and transcripts (**A**) and the percentage of each OTU (97% sequence similarity) in the corresponding clone library (**B**) from the South China Sea *T. swinhoei*. OTU representatives are marked. Scale bar represents 10% nucleotide sequence divergence per homologues position. Bootstrap values more than 50% of 1000 replicates are shown on the tree. The outgroup is an archaeal 16S rRNA sequence of candidatus *Nitrosopumilus* sp. DDS1 (accession no. KR737579).

**Figure 3:**
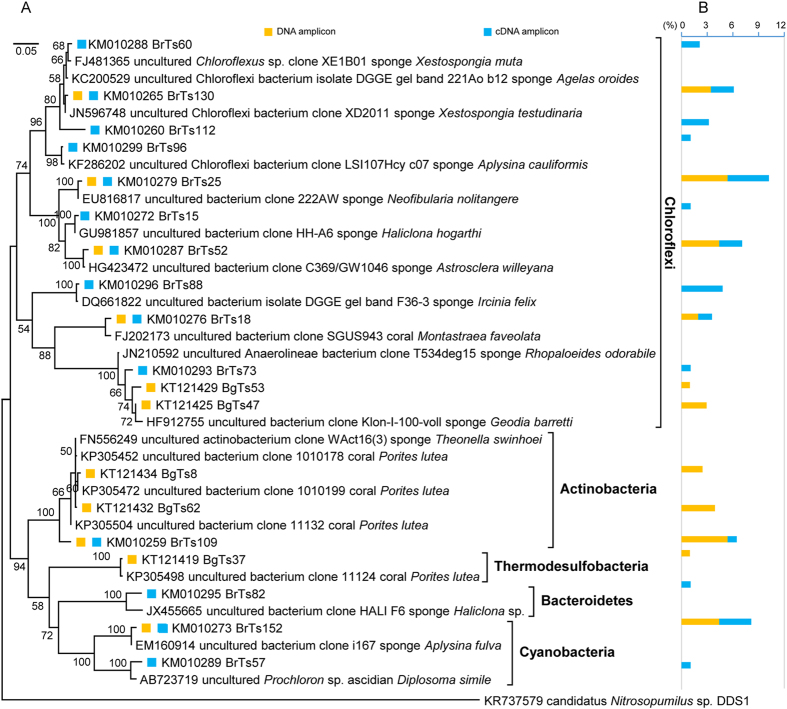
Maximum-likelihood phylogenetic analysis of Chloroflexi, Actinobacteria, Thermodesulfobacteria, Bacteroidetes and Cyanobacteria 16S rRNA genes and transcripts (**A**) and the percentage of each OTU (97% sequence similarity) in the corresponding clone library (**B**) from the South China Sea *T. swinhoei*. OTU representatives are marked. Scale bar represents 5% nucleotide sequence divergence per homologues position. Bootstrap values more than 50% of 1000 replicates are shown on the tree. The outgroup is an archaeal 16S rRNA sequence of candidatus *Nitrosopumilus* sp. DDS1 (accession no. KR737579).

**Figure 4:**
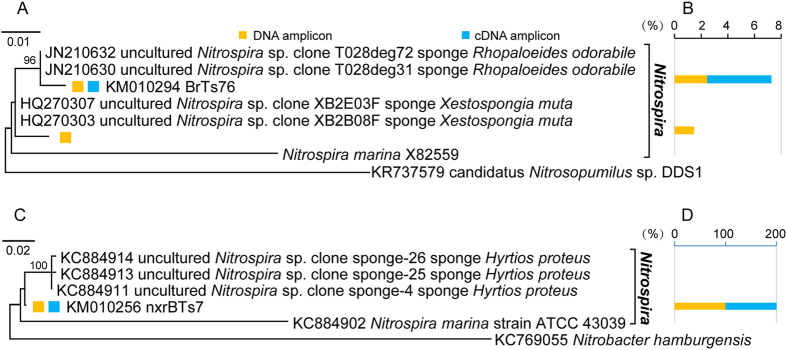
Maximum-likelihood phylogenetic analysis of *Nitrospira* 16S rRNA genes and transcripts (**A**) and the percentage of each OTU (97% sequence similarity) in the corresponding clone library (**B**), and maximum-likelihood phylogenetic analysis of *Nitrospira nxrB* genes and transcripts (**C**) and the percentage of this OTU (95% sequence similarity) in the corresponding clone library (**D**) from the South China Sea *T. swinhoei*. OTU representatives are marked. Scale bar represents 1% (for 16S rRNA) or 2% (for *nxrB*) nucleotide sequence divergence per homologues position. Bootstrap values more than 50% of 1000 replicates are shown on the tree. The outgroup of *Nitrospira* 16S rRNA tree is an archaeal 16S rRNA sequence of candidatus *Nitrosopumilus* sp. DDS1 (accession no. KR737579) and the outgroup of *Nitrospira nxrB* tree is a *nxrB* sequence of *Nitrobacter hamburgensis* (accession no. KC769055).

Bacteria showed higher diversity than archaea in the South China Sea *T. swinhoei*, since 66 bacterial 16S rRNA OTUs were revealed (Figs 2A,3A and 4A; Supplementary Table S3). These OTUs interspersed among 12 reported phyla according to the RDP Classifier identification (Supplementary Table S1) and phylogenetic analysis (Figs 2A,3A and 4A; Supplementary Fig. S2A). Each of these OTUs accounted for 0.5–7.8% of the sequences in the corresponding clone library (Figs 2B,3B and 4B; Supplementary Fig. S2B). In detail, 14 OTUs interspersing among Acidobacteria, Actinobacteria, Chloroflexi, Cyanobacteria, Firmicutes, Nitrospirae, Poribacteria and Proteobacteria were detected at DNA/transcript levels (Supplementary Table S3). Twenty-six OTUs belonging to Acidobacteria, Bacteroidetes, Chloroflexi, Firmicutes, Gemmatimonadetes, Poribacteria, Proteobacteria and Spirochaetes were uniquely revealed at the transcript level, and 28 OTUs falling into Acidobacteria, Actinobacteria, Chloroflexi, Nitrospirae, Proteobacteria, Spirochaetes and Thermodesulfobacteria were uniquely identified by their 16S rRNA genes (Supplementary Table S3). Overall, 40 of 66 OTUs were revealed at the transcript level and fell into 12 reported phyla, indicating diverse bacteria with transcriptional activity in this sponge.

Particularly, one shared 16S rRNA OTU at DNA/transcript levels (accession no. KM010294) and one unique 16S rRNA OTU at the DNA level (accession no. KT121415) fell into the *Nitrospira* genus of Nitrospirae (Fig. 4A; Supplementary Table S1). These OTUs were most similar to the sequences (99% sequence identity) from the sponges *Rhopaloeides odorabile* (accession no. JN210630 and JN210632) and *Xestospongia muta* (accession no. HQ270303 and HQ270307). Meanwhile, none of the Proteobacteria and Chloroflexi 16S rRNA OTUs was ascribed into the AOB genera *Nitrosomonas*, *Nitrosococcus*, *Nitrosospira* or *Nitrosovibrio*, or the NOB genera *Nitrobacter*, *Nitrospina*, *Nitrococcus*, *Nitrospina*, *Nitrotoga* or *Nitrolancea*, according to the phylogenetic analysis and RDP classification (Figs 2A and 3A; Supplementary Table S1). This result indicated that the *Nitrospira* species might dominate the nitrifying bacterial community in the South China Sea *T. swinhoei*.

The presence and activity of NOB in the South China Sea *T. swinhoei* was verified by positive PCR detection of *Nitrospira nxrB* genes and their transcripts. All the *Nitrospira nxrB* gene and transcript amplicons were clustered into one shared OTU at DNA/transcript levels (accession no. KM010256) belonging to the *Nitrospira* cluster (Fig. 4C,D). No other NOB genera were revealed by *nxrA* or *nxrB* PCR assays. This OTU was most similar (98% sequence identity) to the sequences from the sponge *Hyrtios proteus* (accession no. KC884911), and it shared 85% identity to the sequence of *Nitrospira marina* strain ATCC 43039 (accession no. KC884902) (Supplementary Table S2).

Collectively, our results on 16S rRNA genes and transcripts suggested that a fraction of bacteria interspersing among 12 reported phyla had transcriptional activity in the South China Sea *T. swinhoei*. Particularly, *Nitrospira* species dominated the nitrifying bacterial community in this sponge. The revealed *Nitrospira nxrB* genes/transcripts and *Nitrospira*16S rRNA genes/transcripts demonstrated that the *Nitrospira* NOB species had transcriptional activity, and they were predominantly responsible for nitrite oxidation in the South China Sea *T. swinhoei*.

### Quantification of *Nitrosopumilus*-like *amoA* and *Nitrospira nxrB* genes and transcripts

**Figure 5:**
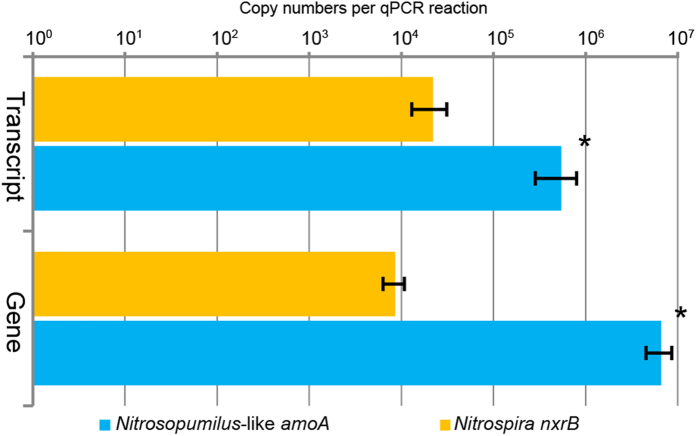
Abundance of *Nitrosopumilus*-like *amoA* genes/transcripts and *Nitrospira nxrB* genes/transcripts estimated by qPCR for the South China Sea *T. swinhoei*. The Mean and standard deviation (SD) of *amoA* and *nxrB* abundances were calculated in Microsoft Excel 2007. T-test was performed using the t-test command of Microsoft Excel 2007 at a significance level of α = 0.05. A statistically significant difference (P < 0.001) between copy numbers of *Nitrosopumilus*-like *amoA* genes and *Nitrospira nxrB* genes, and *Nitrosopumilus*-like *amoA* transcripts and *Nitrospira nxrB* transcripts is indicated with an asterisk.

qPCR assays were employed to quantify the abundance of *Nitrosopumilus*-like *amoA* and *Nitrospira nxrB* genes and their transcripts in the South China Sea *T. swinhoei*. Copy numbers of *Nitrosopumilus*-like *amoA* genes and their transcripts were (6.59 ± 1.21) × 10^6^ and (5.41 ± 2.57) × 10^5^ per reaction, respectively; while copy numbers of *Nitrospira nxrB* genes and their transcripts were (8.55 ± 2.24) × 10^3^ and (2.20 ± 0.91) × 10^4^ per reaction, respectively (Supplementary Table S4). At a significance level of α = 0.05, the abundance of *Nitrosopumilus*-like *amoA* genes was higher than that of *Nitrospira nxrB* genes (P < 0.001, independent-sample t-test) in *T. swinhoei*, which was similar to the transcript-level revelation of this sponge that the abundance of *Nitrosopumilus*-like *amoA* transcripts was higher than that of *Nitrospira nxrB* transcripts (P < 0.001, independent-sample t-test) (Fig. 5). This finding suggested the higher abundances of nitrifying genes and transcripts of *Nitrosopumilus*-like AOA than those of *Nitrospira* NOB in the South China Sea *T. swinhoei*.

## Discussion

Although some lineages, like AOB, retain an appreciable abundance of rRNAs even in the dormant period when their activity is expected to be minimal^41^, it is widely accepted that transcriptional expressions of rRNA genes are typically correlated with cellular growth rate and activity in microbes^42^. The RNA-based analysis of rRNA or functional genes has been successfully used to investigate the activity of sponge symbiotic communities. For example, active microbial community including *Nitrosopumilus*, *Nitrosococcus* and *Nitrospira* nitrifiers, in the sponge *Rhopaloeides odorabile*, showed significant shifts under changed thermal stresses by 16S rRNA assays^43^. Meanwhile, PCR assays have uncovered diverse *Nitrosospira* AOB *amoA* genes but no transcript counterparts in the sponges *Ircinia strobilina* and *Mycale laxissima*^24^. Thus, RNA-based identification of rRNAs or functional genes is a useful means to reveal the metabolically active microbial populations in sponges.

The shared OTUs at DNA/transcript levels, including five *amoA* OTUs, one *nxrB* OTU, four archaeal 16S rRNA OTUs and 14 bacterial 16S rRNA OTUs, indicated the existence of community-wide transcriptionally active prokaryotic populations, especially the nitrifying microbes in the South China Sea *T. swinhoei*. The unique OTUs at the DNA level, including one archaeal 16S rRNA OTUs and 28 bacterial 16S rRNA OTUs, implied that the microbes represented by these OTUs might be during their dormant period or be transcribed at low level which was below the PCR sensitivity threshold. Meanwhile, 26 bacterial 16S rRNA OTUs were uniquely revealed at the transcript level, indicating that these bacterial members may have very low genomic abundance but high transcriptional activity in the South China Sea *T. swinhoei*. Similar DNA- and transcript-level discrepancies of rRNA or functional genes have also been discovered in various sponge species^24^,^32^,^35^, indicating the common existence of selective activity of microbial community in their hosts. Consequently, a large fraction of active archaea and bacteria were suggested by rRNA-based findings, while the active nitrifying community dominated by *Nitrosopumilus*-like AOA and *Nitrospira* NOB were revealed by functional transcript-based assays. This study provides direct molecular evidences for the inhabitancy of nitrifying community with transcriptional activity in the South China Sea *T. swinhoei*.

In contrast to Palauan *T. swinhoei*^21^, no AOB was detected in the South China Sea *T. swinhoei* in this study, suggesting that archaeal not bacterial ammonia oxidizers dominate the first step in nitrification in this sponge, which is consistent with the previous reports on the sponge *Rhopaloeides odorabile* adults and larvae, and other sponges^5^,^43^. The *Nitrospira* bacteria, known to be able to catalyze the nitrite oxidation step, were active in the South China Sea *T. swinhoei*, which is consistent with the revelation of the sponges *Stylissa carteri* and *Geodia barretti*^37^,^38^. No other types of bacteria with recognized capability to oxidize nitrite were detected by 16S rRNA or *nxrA*/*nxrB* targeting, indicating the strict nitrifying bacteria in the South China Sea *T. swinhoei*.

Quantitative assays on nitrifying genes or transcripts have reflected the abundance and transcriptional activity of nitrifying communities in sponges. For example, quantification of *amoA* genes/transcripts has indicated that AOA greatly outnumbered AOB in the sponges *Halisarca caerulea*, *Higginsia thielei* and *Nodastrella nodastrella*^34^. Meanwhile, the abundance of AOA *amoA* transcripts would be significantly affected by the physiological status of sponge *Xestospongia muta*^44^. In this study, qPCR assays revealed higher abundances of nitrifying genes and transcripts of *Nitrosopumilus*-like AOA than those of *Nitrospira* NOB in the South China Sea *T. swinhoei*. Within the nitrification process, the oxidation of ammonia to nitrite is the rate-limiting step, and archaeal ammonia oxidation rates have been reported to be correlated with AOA abundance in the ocean^45^. Therefore, higher abundance of AOA would contribute to the timely removal of the excreted ammonia from sponge to keep the host healthy. Meanwhile, the fact of nitrite oxidation rates exceeding ammonia oxidation rates in the ocean has been verified in the former case^46^. Therefore, higher abundance of AOA than NOB would be necessary in the nitrification process in the investigated *T. swinhoei* samples.

As a ubiquitous sponge species in the Indo-Pacific Ocean^39^, *T. swinhoei*-mediated nitrogen cycle has been partially predicted by the genomic information of the bacterial isolates from *T. swinhoei*. For example, genomic analyses of *T. swinhoei*-derived candidatus *Synechococcus spongiarum* and candidatus *Entotheonella* sp. have revealed the genes related with nitrogen fixation, nitrate reduction, ammonia/nitrite/urea assimilation and ammonia remineralization^47^,^48^. Meanwhile, genomic potentials of ammonia oxidation of *Nitrosopumilus* and *Nitrosospira*, and that of nitrite oxidation of *Nitrospira* have been suggested for Palauan *T. swinhoei* by *amoA* and 16S rRNA genes assays^21^,^40^. In this study, by comparing *amoA*/*nxrB*/16S rRNA genes with their transcripts, we are able to provide a community-wide profile of the *in situ* active microbes, including *Nitrosopumilus*-like AOA and *Nitrospira* NOB in the South China Sea *T. swinhoei*, thus gaining a picture of the active nitrification potentials in this sponge.

Archaea and bacteria have been previously revealed by 16S rRNA gene targeting for Palauan *T. swinhoei*^21^,^40^. Comparison of archaeal 16S rRNA sequences between Palauan *T. swinhoei* and the South China Sea *T. swinhoei* indicated that, archaeal community in these allopatric *T. swinhoei* were dominated by the *Nitrosopumilus* genus. ClustalW sequence alignment analysis (http://www.genome.jp/tools/clustalw/) indicated that, not all of the archaeal 16S rRNA sequences from these allopatric *T. swinhoei* were overlapped, as multiple alignment similarity of these archaeal 16S rRNA fragments is 95.6% (coverage region is archaeal 16S rRNA V3-V5 region, coverage value is 100%) (Supplementary Fig. S3A). Meanwhile, eight bacterial phyla, including Chloroflexi, Proteobacteria, Cyanobacteria, Actinobacteria, Acidobacteria, Nitrospirae, Bacteroidetes and Spirochaetes, were detected in these allopatric *T. swinhoei*; while four phyla, including Firmicutes, Thermodesulfobacteria, Gemmatimonadetes and Poribacteria were only identified from the South China Sea *T. swinhoei*. Particularly, ClustalW sequences alignment analysis indicated that, the *Nitrospira* 16S rRNA fragments from these allopatric *T. swinhoei* showed significant distinction, as multiple alignment similarity of these *Nitrospira* 16S rRNA sequences is 98.6% (coverage region is bacterial 16S rRNA V3 region, coverage value is 100%) (Supplementary Fig. S3B). These revelations indicated the different structures of archaeal and bacterial communities, including AOA and NOB lineages, between these allopatric *T. swinhoei*. Since samples of the South China Sea *T. swinhoei* and Palauan *T. swinhoei* were collected from separate biogeographic regions, different environmental factors, such as temperature, substrate concentration or dissolved oxygen, might lead to the composition shifts of archaeal and bacterial communities between these allopatric *T. swinhoei*. Meanwhile, some microbes that colonize their sponge host could be filtered in from local surrounding seawater, which could also affect the community structure of sponge symbionts^49^.

In this study, PCR cloning and Sanger sequencing methods have been used to reveal the putatively active nitrifying community in the South China Sea *T. swinhoei*. These methods are acceptable in conducting censuses of environmental microbes at the current time. For example, PCR and qPCR analyses of archaeal *amoA* gene have revealed the AOA diversity and abundance in Qinghai Lake^50^. Similar assays have revealed the composition and abundance shifts of archaeal *amoA* transcript in the deep sea^51^. Meanwhile, applications of high-throughput sequencing technologies have expanded the information about community memberships and diversity in various ecosystems. The majority of microbial ecology studies apply high-throughput sequencing by focusing on either gene-targeted or shotgun sequencing^25^. Gene-targeted high-throughput sequencing can provide functional gene information from microbial communities; however, there are several challenges associated with this approach. First, widespread lack of sequence conservation across functionally homologous genes can make PCR primer design difficult, leading to lack of detection of relevant functional genes in the environment. Second, even though fairly conserved primers can be designed for some functional genes of interest, the success of amplification is habitat/ecosystem dependent, most likely due to variations in the quality of extracted DNA, community complexity, sequence divergence, and target gene abundance^25^. Shotgun high-throughput sequencing can avoid many of the biases encountered in amplicon sequencing because it does not require amplification prior to sequencing. While it often fails to provide sufficient sequence depth to assemble the genomes of individual species, especially in complex microbial communities^25^. Another obstacle to adequate sequence coverage is contaminant DNA or RNA, particularly in host-associated microbiome studies, where sequence data may be predominantly from the host^38^. Shotgun-based approaches can also be impaired by dominant populations in the sample, which may be excessively oversampled^38^. In metatranscriptomic studies, this issue can be compounded by high rRNA abundance^37^. Data analysis can be challenging for the high-throughput sequencing technologies, particularly the shotgun sequencing data, as the analysis and assembly of large sequencing data sets are often computationally demanding^52^. In addition, statistical analysis of large short read datasets depends on the length of the reads and the availability of representative reference genomes^25^. Therefore, in this study, PCR-targeted cloning and Sanger sequencing approaches are reliable in specifically elucidating certain ecological function, i.e. the nitrification process in the South China Sea *T. swinhoei*.

## Conclusions

In summary, the composition and activity of nitrifying community in the South China Sea *T. swinhoei* were revealed by targeting 16S rRNA genes, *amoA* and *nxrB* genes and their transcripts. Our investigation demonstrated the inhabitancy and transcriptional activity of *Nitrosopumilus*-like AOA and *Nitrospira* NOB that dominate the nitrifying community in this sponge host. Meanwhile, quantitative assessments indicated relative higher abundances of nitrifying gene and transcript of *Nitrosopumilus*-like AOA than those of *Nitrospira* NOB in this sponge. These results replenished an growing data pool of associations between sponge and nitrifiers, and further contributed to the understanding of the ecological functions of so far uncultivated functional community in sponges.

## Materials and Methods

### Sample collection

*T. swinhoei* was collected by Scuba diving near Xisha Archipelago (16°50′N; 112°20′E) in the South China Sea at a depth of *ca*. 20 m in May, 2009. The sponge species was identified by morphological characterization and 28S rRNA Sequencing. The 28S rRNA gene from this sponge showed 99% similarity to *T. swinhoei* voucher NCI218 28S rRNA gene (accession no. KC884844) and was submitted to GenBank under the accession number JF506040. Samples from three different individuals were cut into 15 mm thick slices, then immediately preserved in RNAfixer (YuanPingHao, Beijing, China) and stored at −70 °C before DNA or RNA extraction.

### RNA, DNA extraction and cDNA synthesis

*T. swinhoei* RNA was extracted using the RNApure Plant Kit (CoWin Biotech, Beijing, China), and DNA was extracted using the DNeasy Plant Mini Kit (Qiagen, Hilden, Germany), respectively, according to the manufacturer’s instructions. RNA and DNA were extracted from the samples of three individuals, respectively; for each individual, several specimens were homogenized to minimize the sampling bias. RNA was treated with DNase (CoWin Biotech) at 37 °C for 30 min and purified with RNA Cleanup Kit (CoWin Biotech). RNA and DNA were accurately quantified by Qubit 2.0 Fluorometer using Qubit RNA BR Assay Kit (Invitrogen, Darmstadt, Germany) and Qubitds DNA BR Assay Kit (Invitrogen), respectively. For reverse-transcription, purified RNA was converted into single strand cDNA with random hexamers primer using the SuperScript First-Strand Synthesis System (Invitrogen, Carlsbad, USA) according to the manufacturer’s protocol. DNA and cDNA products were stored at −70 °C for subsequent experiments.

### Clone library construction

Reported primers (Table 1) targeting *amoA*, *nxrA*, *nxrB* and 16S rRNA gene fragments were selected for PCR-screening from *T. swinhoei* DNA and cDNA templates. A novel primer pair NpnnxrBF/NpnnxrBFR specially targeting the *Nitrospina nxrB* gene was designed using the CODEHOP tool^53^ in this research. *nxrB* sequences of *Nitrospina gracilis*3/211 (accession no. KC262217), *Nitrospina* sp. AB-629-B18 (accession no. A3QC_RS0100660) and an uncultured *Nitrospina* sp. clone (accession no. KJ571877) were selected for primer design to cover a 425 bp region. Forward primer NpnnxrBF and reverse primer NpnnxrBR target the 685–707 and the 1089–1109 nucleotide regions of *nxrB2* gene in *Nitrospina gracilis*3/2, respectively. Specificity of this primer pair targeting *Nitrospina nxrB* was tested by BLASTn (www.ncbi.nlm.nih.gov/blast/) searches against the GenBank database. PCR mixture (40 μl) consists of 2 μl cDNA or 4 ng DNA templates, 0.1 μM of each primer and 20 μl TaqMaster Mix (CoWin Biotech). PCR amplifications were carried out on a Thermocycler (Eppendorf, Hamburg, Germany) according to the following procedures: 95 °C for 5 min; followed by 30 cycles consisting of 95 °C for 40 s, annealing (temperature referring to Table 1) for 1min and 72 °C for 1min; and finally 72 °C for 15 min. For negative control, similar procedure was carried out using purified RNA to ensure that there was no genomic DNA contamination. Triplicate PCR products were pooled to reduce amplification bias and determined by 1.5% agarose gel electrophoresis. PCR products were gel-purified with MinElute Gel Extraction Kit (Qiagen), then ligated into pUC-T vectors (CoWin Biotech) and transformed into *Escherichia coli* DH5α competent cells (CoWin Biotech) according to the standardized protocols. For each gene marker, individual triplicates-derived clones were screened based on ampicillin resistance; while inserts were identified by PCR with vector-specific M13 primers and read on an ABI 3100 capillary sequencer (Sangon Corp., Sequencing Service, Shanghai, China).

### Phylogenetic analysis

The obtained 16S rRNA sequences were checked for chimeras by DECIPHER’s Find Chimeras web tool^54^. All the obtained nucleotide sequences were trimmed manually by using ClustalW implemented in MEGA 6 with default settings. The trimmed sequences were used to perform BLASTn searches against available sequences in the GenBank. Operational taxonomic unit (OTU) was defined as sequence group in which nucleotide sequences differed by 3% for 16S rRNA genes and by 5% for functional genes^55^, respectively, by using the Mothur package^56^. Individual triplicates-derived clones of each gene marker were simultaneously sequenced to make one rarefaction curve and sequencing of clones would be sufficient when the rarefaction curves reach saturation at 3% or 5% nucleotide cutoff value using Mothur package^56^. The 16S rRNA sequences were assigned into different taxa using the RDP Classifier tool^57^. One representative sequence from each OTU and its closest sequence(s) retrieved from GenBank were aligned by using ClustalW implemented in the MEGA 6 for constructing phylogenic trees. Maximum-likelihood tree was constructed by using the MEGA 6 with the Kimura-2 parameter model according to a published guideline^58^. Bootstrap analysis was used to estimate the reliability of phylogenetic reconstructions (1000 replicates). Representative of each OTU was submitted to GenBank under the accession numbers: KM010251 - KM010255 for *amoA* sequences, KM010256 for *nxrB* sequence, KM010247 - KM010250, KM010257 - KM010299, KM121407 - KM121435 and KT380844 for 16S rRNA sequences.

### qPCR assays

qPCR were performed using an ABI 7500 Fast Real-time PCR platform (Applied Biosystems, Foster, CA, USA), according to the procedures used by Radax *et al.*^33^. Three independent biological replicates were performed for each sample with three technical replicates each. For *Nitrosopumilus*-like *amoA* gene, *Nitrospira nxrB* gene, or their transcripts, the PCR was performed in a total volume of 25 μl containing 12.5 μl of SYBR Premix Ex Taq™ II (Takara, Dalian, China), 2ng of DNA template or 1μl of cDNA template and 0.1 μM of each primer (Table 1). The qPCR thermocycling steps were set as follows: 95 °C for 5 min and 40 cycles at 95 °C for 45 s, 57 °C (for *amoA*) or 56 °C (for *nxrB*) for 45 s, and 72 °C for 45 s. Cycling was followed by a final elongation step at 72 °C for 10 min. Standard curves (log-linear R^2^ > 0. 99) were generated using purified and quantified plasmids containing *amoA* sequence (168bp) or *nxrB* sequence (179bp) in a dilution series that spanned from 10^1^ to 10^7^ gene copies per reaction. Plasmid DNA was extracted with the PurePlasmid 96 Kit (CoWin Biotech), and the plasmid concentration was measured with a Qubit 2.0 Fluorometer using Qubitds DNA BR Assay Kit (Invitrogen). Since the sequences of the vector and PCR insert are known, we calculated the copy numbers of *amoA* or *nxrB* directly from the concentration of extracted plasmid DNA according to the reported formula^59^: copy numbers μl^−1^ = (A × 6.022 × 10^23^) × (660 × B)^−1^, where A is the plasmid concentration (g μl^−1^), B is the recombinant plasmid length (bp) containing the *amoA* or *nxrB* sequence, 6.022 × 10^23^ is the Avogadro’s number and 660 is the average molecular weight of one bp. For negative control, similar procedure was carried out using purified RNA to ensure that there was no genomic DNA contamination. After real-time PCR assay, the specificity of amplification was verified by generation of melting curves and agarose gel electrophoresis. Data acquisition and analysis of the Real-time PCR assay were performed using the 7500 System SDS Software Version 1.2 (Applied Biosystems). Data statistics and independent-sample t-test were performed using corresponding commands in Microsoft Excel 2007.

## Additional Information

**How to cite this article**: Feng, G. *et al.* Inhabitancy of active *Nitrosopumilus*-like ammonia-oxidizing archaea and *Nitrospira* nitrite-oxidizing bacteria in the sponge *Theonella swinhoei*. *Sci. Rep.* **6**, 24966; doi: 10.1038/srep24966 (2016).

## Supplementary Material

Supplementary Information

## Figures and Tables

**Table 1:** Primers for the PCR-screen of nitrifying functional genes and 16S rRNA sequences and their amplifying results.

Primer	Sequence (5′-3′)	Target gene	Ta (°C)	Approach	No. of DNA clones	No. of transcript clones	Reference
340F	CCCTAYGGGGYGCASCAG	Archaea 16S rRNA	57	PCR	69	65	9
1000R	GGCCATGCACYWCYTCTC						
356F	ACWCCTACGGGWGGCWGC	Bacteria 16S rRNA	60	PCR	204	187	15
1064R	AYCTCACGRCACGAGCTGAC						
amo111F	TTYTAYACHGAYTGGGCHTGGACATC	Archaea *amoA*	55	PCR	73	68	8
amo643R	TCCCACTTWGACCARGCGGCCATCCA						
archamoAqF	CCRGTCTGGTTRCCDTCAGG	Archaea *amoA*	57	qPCR	–	–	This study
archamoAqR	CTTGAAYGCVGTYTCAAGCG						
amoA1F	GGGGTTTCTACTGGTGGT	Bacteria *amoA*	50	PCR	N.A.	N.A.	12
amoA2R	CCCCTCKGSAAAGCCTTCTTC						
amoA3F	GGTGAGTGGGYTAACMG	Bacteria *amoA-amoB*	45	PCR	N.A.	N.A.	11
amoB4R	GCTAGCCACTTTCTGG						
nxrA1F	CAGACCGACGTGTGCGAAAG	*Nitrobacter*/*Nitrococcus nxrA*	55	PCR	N.A.	N.A.	14
nxrA2R	TCYACAAGGAACGGAAGGTC						
nxrA0038F	GCCAGTGGGAAGAGTTCTATA	*Nitrolancea nxrA*	68	PCR	N.A.	N.A.	13
nxrA3595R	GCCACGTGCGTGTCCCGSGT						
NpnnxrBF	TAYATGTGGTGGAACAAYGTRG	*Nitrospina nxrB*	54	PCR	N.A.	N.A.	This study
NpnnxrBR	GCCTTKCGMGGACAMGCCGC						
nxrB169F	TACATGTGGTGGAACA	*Nitrospira nxrB*	55	PCR	16	27	10
nxrB638R	CGGTTCTGGTCRATCA						
nxrBqF	TGTGGTGGAACAACGTGGAA	*Nitrospira nxrB*	56	qPCR	–	–	This study
nxrBqR	CCCGGCATCGAAAATGGTCA						

N.A., PCR detection was not available; “–”, clone sequencing was not performed.
