# Supplementary material for: Inhabitancy of active Nitrosopumilus-like ammonia-oxidizing archaea and Nitrospira nitrite-oxidizing bacteria in the sponge Theonella swinhoei
Source: Sci Rep. 2016 Apr 26;6:24966. doi: 10.1038/srep24966 (PMC4844951; doi:10.1038/srep24966)
Supplement: Supplementary Information [file srep24966-s1.pdf]

Supplementary information

Inhabitancy of active *Nitrosopumilus*-like ammonia-oxidizing archaea and *Nitrospira* nitrite-oxidizing bacteria in the sponge *Theonella swinhoei*

Guofang Feng, Wei Sun, Fengli Zhang, Loganathan Karthik and Zhiyong Li*

*** Correspondence:** Zhiyong Li: zyli@sjtu.edu.cn

Table S1 RDP rRNA Classifier assignment of 16S rRNA OTUs (97% sequence similarity) revealed from the South China Sea *T. swinhoei*

ID _OTU	RDP rRNA Classifier assignment Detail (Confidence threshold: 50%)
**Archaeal 16S rRNA**	
KM010247_ArTs3	Thaumarchaeota[100%] Nitrosopumilales[100%] Nitrosopumilaceae[100%] Nitrosopumilus[100%]
KM010248_ArTs17	Thaumarchaeota[100%] Nitrosopumilales[100%] Nitrosopumilaceae[100%] Nitrosopumilus[100%]
KM010249_ArTs44	Thaumarchaeota[100%] Nitrosopumilales[100%] Nitrosopumilaceae[100%] Nitrosopumilus[100%]
KM010250_ArTs62	Thaumarchaeota[100%] Nitrosopumilales[100%] Nitrosopumilaceae[100%] Nitrosopumilus[100%]
KT380844_ArTs15D	Thaumarchaeota[100%] Nitrosopumilales[100%] Nitrosopumilaceae[100%] Nitrosopumilus[100%]
**Bacterial 16S rRNA**	
KM010281_BrTs32	Acidobacteria[93%] Acidobacteria_Gp21[93%] Gp21[93%]
KM010278_BrTs21	Acidobacteria[25%] Acidobacteria_Gp1[12%] Candidatus Koribacter[11%]
KT121413_BgTs27	Acidobacteria[100%] Acidobacteria_Gp11[100%] Gp11[100%]
KT121433_BgTs72	Acidobacteria[71%] Acidobacteria_Gp10[71%] Gp10[71%]
KT121410_BgTs17	Acidobacteria[76%] Acidobacteria_Gp6[72%] Gp6[72%]
KT121428_BgTs5	Acidobacteria[97%] Acidobacteria_Gp6[97%] Gp6[97%]
KM010259_BrTs109	Actinobacteria[95%] Actinobacteria[94%] Acidimicrobidae[57%] Acidimicrobiales[57%] Acidimicrobineae[57%] Acidimicrobineae_incertae_sedis[31%] Aciditerrimonas[31%]
KT121434_BgTs8	Actinobacteria[100%] Actinobacteria[100%] Acidimicrobidae[87%] Acidimicrobiales[87%] Acidimicrobineae[87%] Acidimicrobiaceae[50%] Ferrimicrobium[38%]
KT121432_BgTs62	Actinobacteria[100%] Actinobacteria[100%] Acidimicrobidae[75%] Acidimicrobiales[75%] Acidimicrobineae[75%] Acidimicrobiaceae[31%] Ferrimicrobium[22%]
KM010295_BrTs82	Bacteroidetes[100%] Cytophagia[70%] Cytophagales[70%] Flammeovirgaceae[66%] Fabibacter[48%]
KM010260_BrTs112	Chloroflexi[46%] Thermoflexia[36%] Thermoflexales[36%] Thermoflexaceae[36%] Thermoflexus[36%]
KM010276_BrTs18	Chloroflexi[99%] Caldilineae[98%] Caldilineales[98%] Caldilineaceae[98%] Litorilinea[93%]
KM010265_BrTs130	Chloroflexi[40%] Thermoflexia[32%] Thermoflexales[32%] Thermoflexaceae[32%] Thermoflexus[32%]
KM010272_BrTs15	Chloroflexi[57%] Thermoflexia[13%] Thermoflexales[13%] Thermoflexaceae[13%] Thermoflexus[13%]
KM010279_BrTs25	Chloroflexi[49%] Ardenticatenia[15%] Ardenticatenales[15%] Ardenticatenaceae[15%] Ardenticatena[15%]
KM010287_BrTs52	Chloroflexi[49%] Thermoflexia[16%] Thermoflexales[16%] Thermoflexaceae[16%] Thermoflexus[16%]
KM010288_BrTs60	Chloroflexi[53%] Thermoflexia[40%] Thermoflexales[40%] Thermoflexaceae[40%] Thermoflexus[40%]
KM010296_BrTs88	Chloroflexi[72%] Dehalococcoidetes[43%] Dehalogenimonas[43%]

KM010299_BrTs96	Chloroflexi[40%] Thermoflexia[23%] Thermoflexales[23%] Thermoflexaceae[23%] Thermoflexus[23%]
KM010293_BrTs73	Chloroflexi[44%] Anaerolineae[44%] Anaerolineales[44%] Anaerolineaceae[44%] Longilinea[42%]
KT121425_BgTs47	Chloroflexi[23%] Anaerolineae[22%] Anaerolineales[22%] Anaerolineaceae[22%] Longilinea[10%]
KT121429_BgTs53	Chloroflexi[20%] Anaerolineae[20%] Anaerolineales[20%] Anaerolineaceae[20%] Longilinea[9%]
KM010273_BrTs152	Cyanobacteria/Chloroplast[100%] Cyanobacteria[100%] Family II[100%] GpIIa[100%]
KM010289_BrTs57	Cyanobacteria/ Chloroplast[100%] Cyanobacteria[100%] Family VIII[60%] GpVIII[60%]
KM010262_BrTs12	Firmicutes[64%] Clostridia[63%] Clostridiales[60%] Incertae Sedis III[49%] Fervidicola[38%]
KM010267_BrTs132	Firmicutes[39%] Clostridia[35%] Natranaerobiales[11%] Natranaerobiaceae[11%] Dethiobacter[11%]
KM010283_BrTs37	Firmicutes[64%] Clostridia[57%] Clostridiales[37%] Incertae Sedis III[17%] Fervidicola[15%]
KM010285_BrTs45	Firmicutes[33%] Clostridia[32%] Clostridiales[28%] Incertae Sedis III[21%] Fervidicola[19%]
KM010290_BrTs59	Gemmatimonadetes[6%] Gemmatimonadetes[6%] Gemmatimonadales[6%] Gemmatimonadaceae[6%] Gemmatimonas[6%]
KM010294_BrTs76	Nitrospirae[90%] Nitrospira[90%] Nitrospirales[90%] Nitrospiraceae[90%] Nitrospira[90%]
KT121415_BgTs3	Nitrospirae[84%] Nitrospira[84%] Nitrospirales[84%] Nitrospiraceae[84%] Nitrospira[84%]
KM010270_BrTs148	Proteobacteria[100%] Alphaproteobacteria[100%] Rhizobiales[83%] Rhodobiaceae[78%] Parvibaculum[44%]
KT121412_BgTs24	Proteobacteria[100%] Alphaproteobacteria[100%] Rhodobacterales[100%] Rhodobacteraceae[100%] Silicibacter[74%]
KM010266_BrTs131	Proteobacteria[100%] Alphaproteobacteria[100%] Rhodobacterales[100%] Rhodobacteraceae[100%] Loktanella[52%]
KM010297_BrTs9	Proteobacteria[100%] Alphaproteobacteria[100%] Sneathiellales[30%] Sneathiellaceae[30%] Oceanibacterium[23%]
KT121430_BgTs55	Proteobacteria[100%] Alphaproteobacteria[100%] Rhodobacterales[100%] Rhodobacteraceae[100%] Oceanicola[45%]
KT121431_BgTs6	Proteobacteria[100%] Alphaproteobacteria[100%] Rhizobiales[57%] Brucellaceae[38%] Mycoplana[19%]
KM010284_BrTs40	Proteobacteria[100%] Alphaproteobacteria[99%] Rhizobiales[37%] Rhodobiaceae[20%] Rhodoligotrophos[20%]
KT121416_BgTs33	Proteobacteria[100%] Betaproteobacteria[100%] Burkholderiales[100%] Comamonadaceae[100%] Acidovorax[100%]
KM010261_BrTs115	Proteobacteria[100%] Gammaproteobacteria[100%] Enterobacteriales[100%] Enterobacteriaceae[100%] Escherichia/ Shigella[100%]
KM010269_BrTs146	Proteobacteria[100%] Gammaproteobacteria[100%] Oceanospirillales[100%] Hahellaceae[100%] Endozoicomonas[100%]
KT121407_BgTs103	Proteobacteria[100%] Gammaproteobacteria[100%] Vibrionales[92%] Vibrionaceae[92%] Photobacterium[90%]
KT121408_BgTs12	Proteobacteria[100%] Gammaproteobacteria[99%] Thiotrichales[30%] Piscirickettsiaceae[29%] Cycloclasticus[28%]
KT121409_BgTs14	Proteobacteria[100%] Gammaproteobacteria[100%] Pseudomonadales[100%] Moraxellaceae[100%] Acinetobacter[100%]

KT121414_BgTs28	Proteobacteria[100%] Gammaproteobacteria[100%] Gammaproteobacteria_incertae_sedis[80%] Thiohalophilus[36%]
KT121411_BgTs2	Proteobacteria[100%] Gammaproteobacteria[100%] Oceanospirillales[100%] Hahellaceae[100%] Endozoicomonas[100%]
KT121418_BgTs203	Proteobacteria[100%] Gammaproteobacteria[100%] Legionellales[92%] Coxiellaceae[92%] Coxiella[92%]
KT121420_BgTs137	Proteobacteria[100%] Gammaproteobacteria[100%] Oceanospirillales[100%] Hahellaceae[100%] Endozoicomonas[100%]
KT121421_BgTs38	Proteobacteria[100%] Gammaproteobacteria[100%] Oceanospirillales[100%] Hahellaceae[100%] Endozoicomonas[100%]
KT121422_BgTs39	Proteobacteria[100%] Gammaproteobacteria[100%] Chromatiales[68%] Chromatiaceae[61%] Thioflavicoccus[37%]
KT121423_BgTs42	Proteobacteria[100%] Gammaproteobacteria[100%] Chromatiales[63%] Chromatiaceae[27%] Thioflavicoccus[21%]
KT121424_BgTs46	Proteobacteria[98%] Gammaproteobacteria[95%] Gammaproteobacteria_incertae_sedis[47%] Methylohalomonas[36%]
KT121426_BgTs107	Proteobacteria[100%] Gammaproteobacteria[100%] Pseudomonadales[100%] Pseudomonadaceae[100%] Pseudomonas[100%]
KT121435_BgTs9	Proteobacteria[100%] Gammaproteobacteria[100%] Oceanospirillales[100%] Halomonadaceae[100%] Halomonas[100%]
KM010277_BrTs20	Proteobacteria[99%] Gammaproteobacteria[95%] Oceanospirillales[44%] Hahellaceae[11%] Kistimonas[10%]
KM010263_BrTs125	Proteobacteria[71%] Deltaproteobacteria[55%] Syntrophobacterales[37%] Syntrophaceae[29%] Desulfomonile[29%]
KM010286_BrTs50	Proteobacteria[46%] Deltaproteobacteria[35%] Desulfuromonadales[20%] Geobacteraceae[8%] Geopsychrobacter[8%]
KM010274_BrTs159	Poribacteria[100%] incertae_sedis[100%]
KM010275_BrTs163	Poribacteria[100%] incertae_sedis[100%]
KM010291_BrTs64	Poribacteria[100%] incertae_sedis[100%]
KM010292_BrTs7	Poribacteria[100%] incertae_sedis[100%]
KM010282_BrTs34	Poribacteria[100%] incertae_sedis[100%]
KM010271_BrTs149	Poribacteria[48%] incertae_sedis[48%]
KM010257_BrTs1	Spirochaetes[39%] Spirochaetia[39%] Spirochaetales[39%] Spirochaetaceae[26%] Spirochaeta[18%]
KT121427_BgTs49	Spirochaetes[9%] Spirochaetia[9%] Spirochaetales[9%] Spirochaetales_incertae_sedis[7%] Exilispira[7%]
KT121419_BgTs37	Thermodesulfobacteria[18%] Thermodesulfobacteria[18%] Thermodesulfobacteriales[18%] Thermodesulfobacteriaceae[18%] Thermosulfurimonas[18%]

Table S2 BLASTn searches of *amoA* and *nxrB* OTUs (95% sequence similarity) revealed from the South China Sea *T. swinhoei* against the NCBI nr/nt database

**ID_OTU**	**Most similar hits by BLASTn identification**	
	**Uncultured sequence/ ID/ host/ similarity/Coverage**	**Cultured sequence/ ID/ similarity/Coverage**
**Archaeal *amoA***		
KM010251_AmoTs9	uncultured crenarchaeote clone LuffGrA / EU049831/ sponge *Luffariella* sp./ 98%/98%	candidatus *Nitrosopumilus* sp. AR2/ CP003843/ 79%/100%; *Cenarchaeum symbiosum* A/ DQ397580/ 79%/100%
KM010252_AmoTs60	uncultured crenarchaeote isolate B14N(1:A2)/ GQ485791/ sponge *Xestospongia muta*/ 93%/100%	*Nitrosopumilus maritimus* isolate SF_AOA_H10/ HM345611/ 81%/100%
KM010253_AmoTs27	uncultured crenarchaeote clone LuffGrA/ EU049831/ sponge *Luffariella* sp./ 99%/100%	*Nitrosopumilus maritimus* SCM1/ CP000866/ 79%; *Cenarchaeum symbiosum* A/ DQ397580/ 79%/100%
KM010254_AmoTs32	uncultured crenarchaeote clone LuffL19/ EU049833/ sponge *Luffariella* sp./ 95%/100%	*Nitrosopumilus maritimus* SCM1/ CP000866/ 80%/100%
KM010255_AmoTs11	uncultured crenarchaeote clone LuffGrB/ EU049832/ sponge *Luffariella* sp./ 97%/100%	*Nitrosopumilus maritimus* isolate SF_AOA_A10/ HM345609/ 81%/100%
Nitrospira nxrB		
KM010256_nxrBTs7	uncultured *Nitrospira* sp. clone sponge-4 KC884911/ sponge *Hyrtios proteus*/ 98%/96%	*Nitrospira marina* strain ATCC 43039/ KC884902/ 85%/100%

Table S3 Number of 16S rRNA OTUs (97% sequence similarity) in corresponding phylum according to the RDP classification

Phylum	No. of DNA-level OTUs in the phylum	No. of transcript-level OTUs in the phylum	No. of DNA-/ transcript-level shared OTUs in the phylum	Total no. of OTUs in the phylum
Thaumarchaeota	4	5	4	5
Acidobacteria	5	2	1	6
Actinobacteria	3	1	1	3
Bacteroidetes	0	1	1	1
Chloroflexi	6	10	4	12
Cyanobacteria	1	2	1	2
Firmicutes	1	4	1	4
Gemmatimonadetes	0	1	0	1
Nitrospirae	2	1	1	2
Proteobacteria	18	11	3	26
Poribacteria	4	6	4	6
Spirochaetes	1	1	0	2
Thermodesulfobacteria	1	0	0	1

Table S4 Gene and transcript copy numbers of *Nitrosopumilus*-like *amoA* and *Nitrospira nxrB* in individual triplicates of the South China Sea *T. swinhoei*.

Target	Biological replicates	Gene copy numbers per qPCR reaction: Technique replicates	Mean	SD	Transcript copy numbers per qPCR reaction: Technique replicates	Mean	SD
Nitrosopumilus-like *amoA*	Individual #1	8.41e+6	6.59e+6	2.05e+6	8.71e+5	5.41e+5	2.57e+5
		8.28e+6			8.59e+5		
		8.37e+6			8.74e+5		
	Individual #2	7.38e+6			4.63e+5		
		7.56e+6			4.68e+5		
		7.61e+6			4.68e+5		
	Individual #3	3.85e+6			2.82e+5		
		3.89e+6			2.91e+5		
		3.94e+6			2.96e+5		
Nitrospira nxrB	Individual #1	8.28e+3	8.55e+3	2.24e+3	2.21e+4	2.20e+4	9.05e+3
		8.33e+3			2.19e+4		
		8.30e+3			2.25e+4		
	Individual #2	1.09e+4			3.26e+4		
		1.11e+4			3.25e+4		
		1.17e+4			3.22e+4		
	Individual #3	6.06e+3			1.11e+4		
		6.12e+3			1.17e+4		
		6.12e+3			1.18e+4		

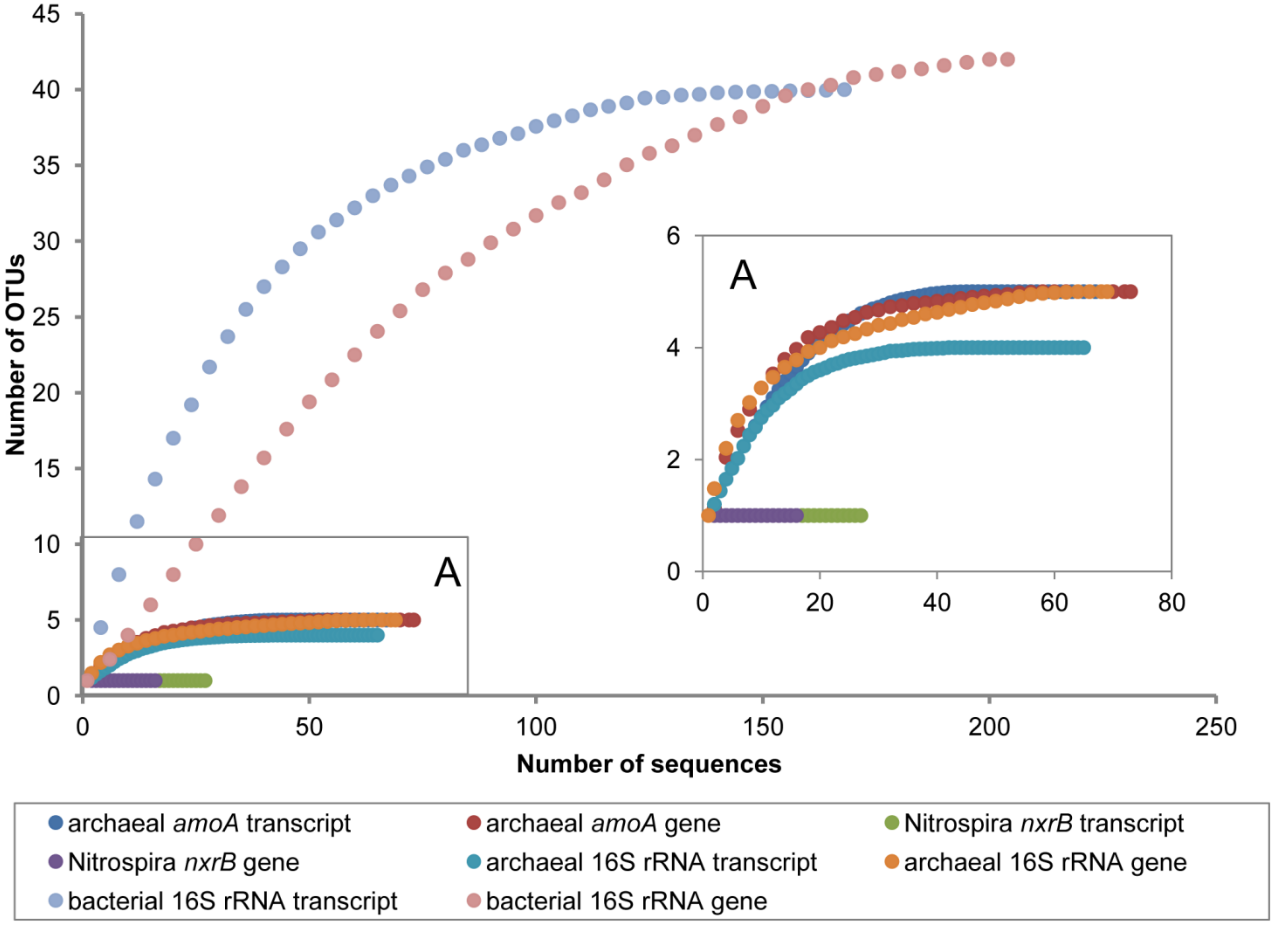

Fig. S1. Rarefaction curves of archaeal and bacterial 16S rRNA genes and transcripts, *Nitrosopumilus amoA* genes and transcripts, *Nitrospira nxrB* genes and transcripts. The curves were analyzed by 95% sequence similarity for *amoA*, *nxrB* sequences and 97% for 16S rRNA sequences.

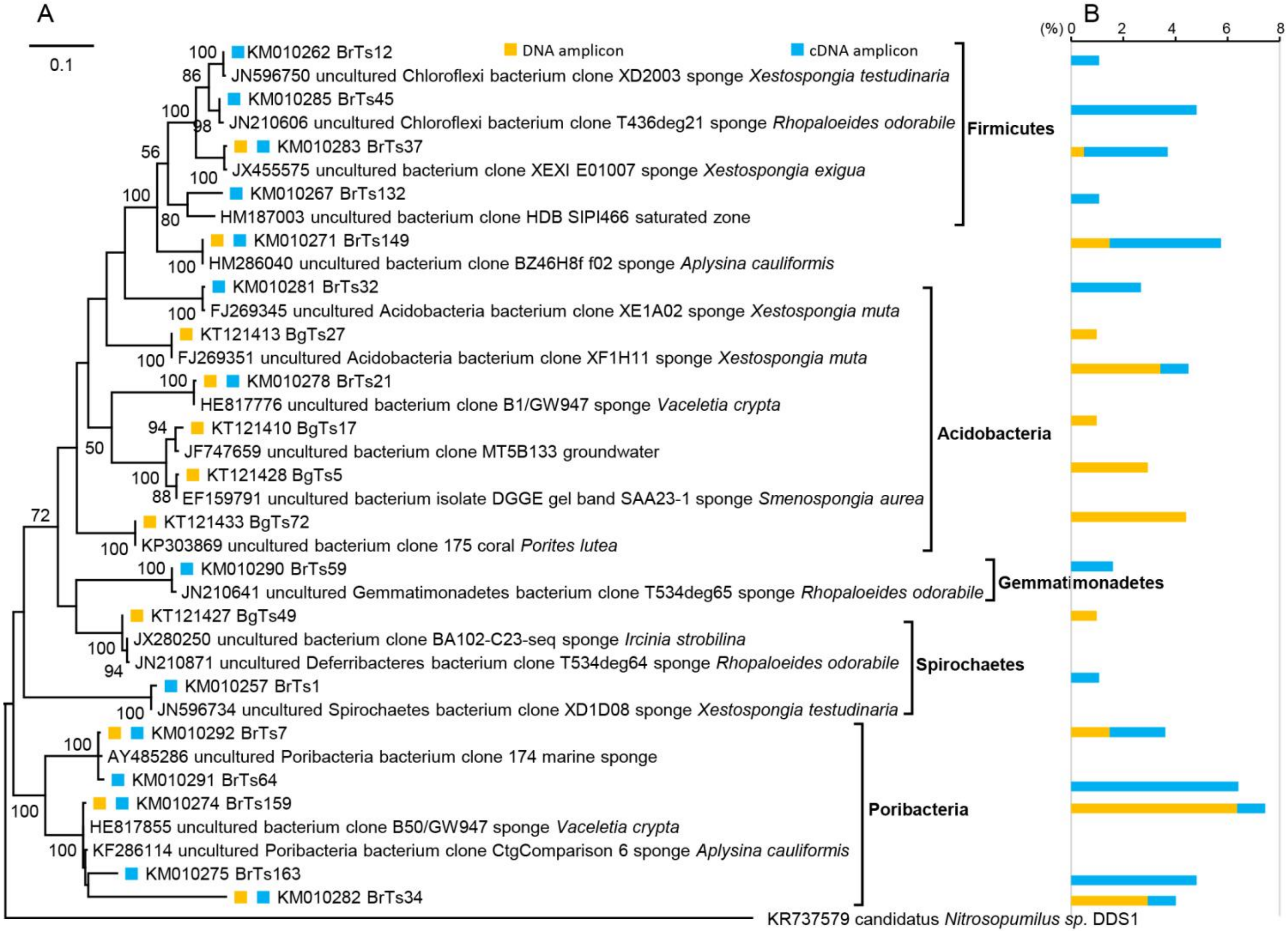

Fig. S2. Maximum-likelihood phylogenetic analysis of Firmicutes, Acidobacteria, Gemmatmonadetes, Spirochaetes, and Poribacteria 16S rRNA gene/transcript OTUs (97% sequence similarity) (A) and their percentage in corresponding clone library (B) from the South China Sea *T. swinhoei*. OTU representatives are marked. Scale bar represents 10% nucleotide sequence divergence per homologues position. Bootstrap values more than 50% of 1000 replicates are shown on the tree. The outgroup was an archaeal 16S rRNA sequence of candidatus *Nitrosopumilus* sp. DDS1 (accession no. KR737579).

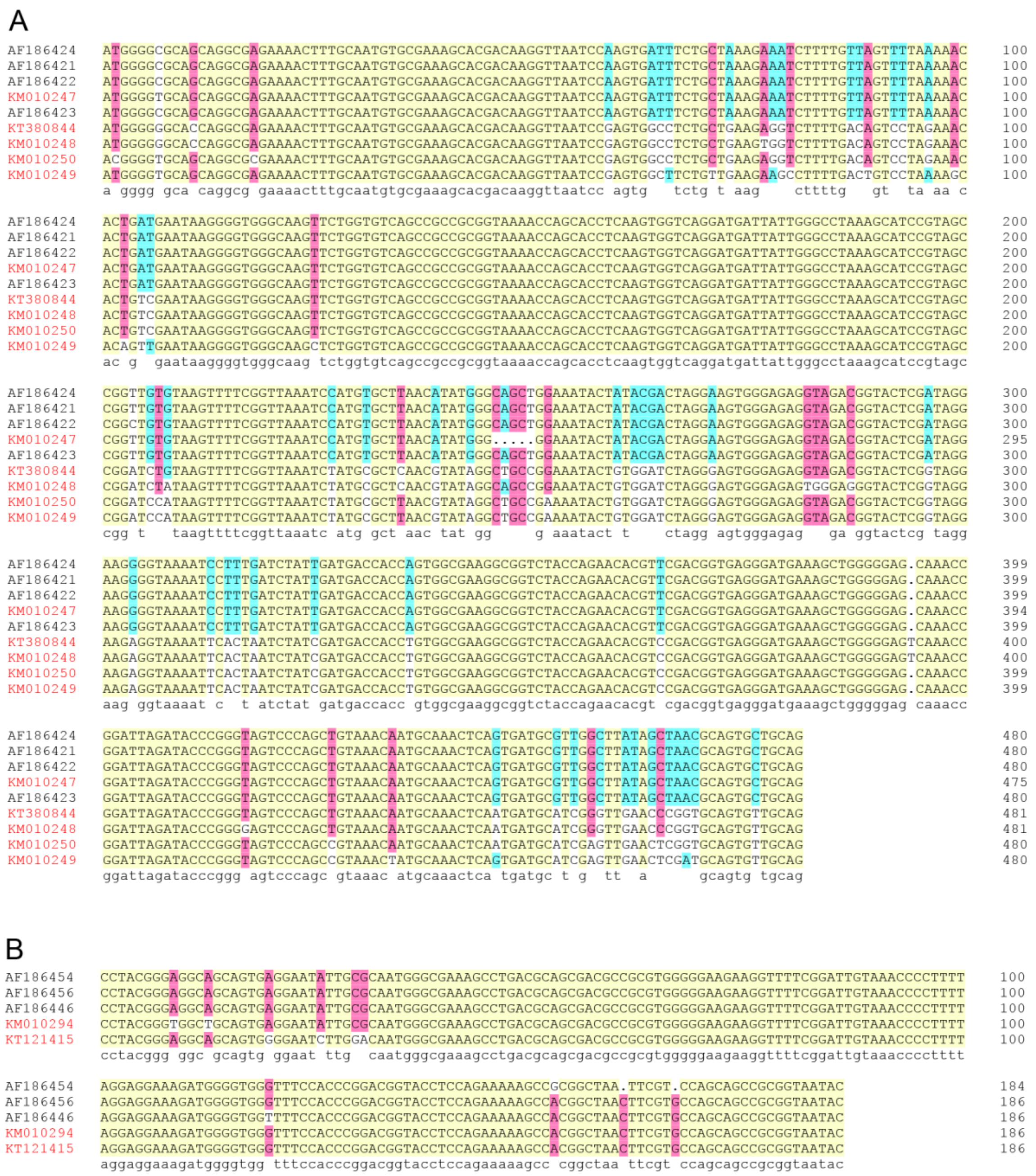

Fig. S3. Sequences alignments of the V3-V5 region of archaeal 16S rRNA sequences from the South China Sea *T. swinhoei* (KM010247 - KM010250, KT380844, red marked) and Palauan *T. swinhoei* (AF186421- AF186424, black marked) (A) and sequences alignments of the V3 region of *Nitrospira* 16S rRNA sequences from the South China Sea *T. swinhoei* (KM010294, KT121415, red marked) and Palauan *T. swinhoei* (AF186446, AF186454, AF186456, black marked) (B). The multiple sequence alignment was performed by ClustalW alignment online tool and alignment visualization used the DNAMAN 6.0
